# The Accessory Protein ORF3 Contributes to Porcine Epidemic Diarrhea Virus Replication by Direct Binding to the Spike Protein

**DOI:** 10.3390/v10080399

**Published:** 2018-07-28

**Authors:** Challika Kaewborisuth, Qigai He, Anan Jongkaewwattana

**Affiliations:** 1 Virology and Cell Technology Laboratory, National Center for Genetic Engineering and Biotechnology (BIOTEC), National Science and Technology Development Agency (NSTDA), Pathumthani 12120, Thailand; challika.kae@biotec.or.th; 2 State Key Laboratory of Agricultural Microbiology, College of Veterinary Medicine, Huazhong Agricultural University, Wuhan 430070, China; he628@mail.hzau.edu.cn

**Keywords:** porcine epidemic diarrhea virus, accessory protein ORF3, spike protein, subcellular localization, virus replication

## Abstract

The porcine epidemic diarrhea virus (PEDV) is an important swine pathogen responsible for severe watery diarrhea, particularly in neonatal piglets. Despite extensive studies performed to elucidate the function of several viral proteins, the contribution of an accessory protein ORF3 in PEDV replication is still largely unknown. Here, we constructed expression plasmids as well as recombinant PEDV carrying myc-tagged ORF3 to assess their expression and subcellular localization in both transfected and infected cells. In PEDV-infected cells, ORF3 was predominantly localized in the cytoplasm, partially in the endoplasmic reticulum (ER) and the Golgi apparatus (Golgi). Interestingly, ORF3 with the N-terminal Flag tag was also detected on the cell surface concomitant with the spike (S) protein as determined by flow cytometry and confocal microscopy. ORF3 and S proteins were also co-localized at perinuclear compartments and in the vesicle-like structures in transfected and infected cells. We also demonstrated that both full-length and naturally truncated ORF3 proteins could interact with the S protein but with different binding affinity, which correlate with the ability of the protein to regulate virus replication in cell culture. Collectively, our results underscore the unprecedented role of the ORF3, which involves the interaction of ORF3 with S and, possibly, other structural protein during PEDV replication.

## 1. Introduction

The porcine epidemic diarrhea virus (PEDV), a member of the family *Coronaviridae*, is an enveloped, single-stranded, positive-sense RNA virus that causes severe enteric diseases with a high mortality rate in suckling piglets [1]. For decades, pathogenic strains of PEDV have caused recurrent outbreaks with enormous economic losses worldwide. The 28-kb PEDV genome has a gene organization typical of an *Alphacoronavirus*, i.e., *ORF 1a/b*, spike (*S*), open reading frame 3 (*ORF3*), envelope (*E*), membrane (*M*) and the nucleocapsid (*N*) genes [2]. Although not entirely elucidated, it is assumed that PEDV replication is initiated by the interaction of the S protein to cellular receptors, followed by endocytosis-mediated viral internalization [3]. After the release of viral genomic RNA into the cytoplasm, replicase proteins undergo extensive intracellular membranes modification to promote viral RNA synthesis. Shortly after synthesis of structural proteins, nascent virions are assembled in the endoplasmic reticulum-Golgi intermediate compartment (ERGIC), before being released, via smooth-walled vesicles, by exocytosis. While the roles of structural proteins in coronavirus (CoV) replication have been extensively studied, the contribution of the accessory ORF3 protein, particularly in PEDV replication, remains quite elusive. Mounting evidence has suggested that ORF3 is one of the key determinants of PEDV virulence [4]. Nevertheless, the mechanisms by which it modulates viral pathogenicity are as yet clearly defined.

The ORF3 protein exhibits a genetic variation among PEDVs. The full-length ORF3 (675 nt; 224 aa) is found in wild-type PEDVs, while the truncated ORF3 (624 nt; 207 aa; 82–98 amino acid deletion) is common in cell-adapted or attenuated PEDVs [4]. Structurally, ORF3 has been predicted to harbor multiple transmembrane domains, existing as a tetrameric ion channel [5]. However, results from assays using cells stably expressing ORF3 revealed that the protein was predominantly expressed in the cytoplasm [6]. Cells expressing ORF3 also displayed a prolonged S phase of the cell cycle and augmented vesicle formation [6]. These results altogether point to the possibility that ORF3 might not be a typical ion channel, but rather a multifunctional protein that is involved in many cellular processes. Notably, since cell culture-adapted variants of PEDV usually possess abortive ORF3, be it absent or truncated [4], it is also likely that ORF3 might interfere with PEDV replication in cultured cells [7].

To date, the investigation of ORF3 is hampered by technical challenges associated with obtaining productively-growing PEDV with intact ORF3. It is also difficult to assess ORF3 expression in virus-infected cells as specific antibodies are either not accessible or ineffective. In this study, we constructed recombinant PEDV bearing ORF3 with specific epitope tags and evaluated the ORF3 expression in infected cells in comparison to those transfected by the expression plasmid. We demonstrated that the expression of ORF3 in PEDV-infected cells is distinct from that transiently expressed in individual cells. Furthermore, we found that ORF3 and S are co-localized in several cellular compartments in infected cells and the ORF3-S interaction was also confirmed. Collectively, our results suggest that ORF3 may interact with S during PEDV assembly and consequently exert its activity at this step of virus replication.

## 2. Materials and Methods

### 2.1. Cell Line and Culture Condition

Human embryonic kidney (HEK) 293T and African green monkey (VeroE6) cells stably expressing porcine aminopeptidase N (VeroE6-APN) were grown and maintained in Opti-MEM medium supplemented with 10% heat-inactivated fetal bovine serum (FBS). Cells were cultured in humidified air containing 5% CO_2_ at 37 °C.

### 2.2. Construction of Expression Plasmids and PEDV Infectious Clones Carrying Tagged-ORF3s

The ORF3 sequence was synthesized for expression in mammalian cells and codon-optimized as shown in Table S1. The full-length (ORF3-FL) and truncated ORF3 (ORF3-Trnc) (deletion of the amino acid at positions 82–98) with myc-tag at the C-terminus were cloned into the pCAGGS expression plasmid, designated pCAGGS_ORF3-FL and-Trnc. The pCAGGS_FlagORF3-FL and-Trnc were also constructed by insertion of Flag-tag at the N terminus of ORF3s.

To generate PEDV infectious clones, the plasmids pCAGGS_ORF3-FL and-Trnc were used as templates for amplification of ORF3-myc flanked with *BsiWI* and *XhoI* restriction sites. The amplified ORF3 variants were cloned into pTZ5R/T carrying *S*, *E*, *M* and *N* genesof PEDV_AVCT12_ at *Bsi*WI and *Xho*I sites to incorporate ORF3 variants into the PEDV_AVCT12_ genome as previously described [8], designated pTZ_ORF3-FL and-Trnc. The pPEDV_AVCT12_ infectious cDNA clone was subsequently digested with *Pac*I and *Mlu*I restriction enzymes to allow the insertion of the ORF3s-myc fragment. PEDV_AVCT12_ genes including S, ORF3-myc, E, M, and the N terminus of the N gene regions in pTZ_ORF3 plasmids were then inserted into the linearized pPEDV_AVCT12_ infectious cDNA clone. The ORF3-myc in positive clones, designated pPEDV_AV12__ORF3-FL and-Trnc, were verified by direct nucleotide sequencing.

### 2.3. Western Blot Assay

HEK293T or VeroE6-APN cells were grown in 6-well plates and transfected with the pCAGGS_ORF3-FL, pCAGGS_ORF3-Trnc or pCAGGS_S_AV12_ using Fugene HD transfection reagent (Promega, Madison, WI, USA) following the manufacturer’s instructions. At 24 h post transfection (hpt), transfected cells were collected and re-suspended in mammalian cell lysis buffer. Fifty micrograms of protein samples were incubated with protein loading buffer at 37 °C for 1 h, loaded on to 10% polyacrylamide gel, and transferred to nitrocellulose membranes. Membranes were then probed with rabbit anti-myc antibodies (Abcam, Cambridge, MA, USA) and followed by IRDye^®^ 800CW goat anti-rabbit IgG (Li-COR bioscience, Lincoln, NE, USA) antibodies. Protein bands were visualized using a Li-COR Odyssey CLx imager (Li-COR bioscience).

### 2.4. Generation of Recombinant Viruses Expressing Tagged-ORF3

VeroE6-APN cells grown in a 6-well plate were transfected with 2 µg of pPEDV_AV12__ORF3-FL or-Trnc and treated with 0.1% recombinant trypsin (Thermo Scientific, Waltham, MA, USA) at 24 hpt. At 60 hpt, cell supernatants were collected and adsorbed onto fresh VeroE6-APN cells to further propagate the virus.

Viral RNA was isolated using an RNA isolation kit (Geneaid, New Taipei, Taiwan). The purified viral RNA was then subject to RT-PCR using PrimeScript™ One-Step RT-PCR Kit (Takara, Tokyo, Japan) using primers specific for the inserted ORF3 gene. PCR products were cloned into pTZ5R/T vector, and five independent clones were sequence verified.

### 2.5. Confocal Microscopy

HEK293T or VeroE6-APN cells were grown on coverslips in 6-well plates. At 24 hpt (or h post infection; hpi), cells were washed with phosphate-buffered saline (PBS) and fixed with 4% paraformaldehyde for 20 min at 4 °C. After incubation, cells were washed three times with PBS and blocked with PBS containing 10% FBS, 1% bovine serum albumin (BSA) and 0.2% TritonX-100 for 1 h. Cells were subsequently incubated for 1 h with rabbit anti-myc antibodies (Abcam) and mouse anti-calreticulin ER marker (Abcam) or mouse anti-58K Golgi protein antibodies (Abcam) in 10% FBS at a dilution 1:500 and 1:250, respectively. After washing thrice with PBST, goat anti-rabbit IgG Alexa flour 488 (Abcam) and goat anti-mouse IgG Alexa flour 647 antibodies (Abcam) in 10% FBS at a dilution 1:1000 was added and further incubated for 1 h. The glass slips were mounted on slides with Prolong Gold Antifade Mountant with DAPI (Invitrogen, Carlsbad, CA, USA). The samples were analyzed by FluoviewTM FV1000 confocal microscopy (Olympus, Tokyo, Japan). The Pearson’s correlation coefficients (PCC, Pasadena, CA, USA) were employed to determine co-localization using the ImageJ analysis program [9] and the PSC co-localization plug-in as described elsewhere [10,11,12]. Mean Pearson’s coefficient values were calculated from at least ten independent acquired images.

### 2.6. Flow Cytometry

HEK293T cells were transfected with pCAGGS_FlagORF3-FL and-Trnc for 24 h before being trypsinized and fixed with 4% paraformaldehyde. Cells were blocked in blocking buffer (2% FBS and 0.5% BSA in PBS) for 1 h and then incubated with rabbit anti-Flag tag antibodies (Abcam) or mouse anti-S1 monoclonal antibodies for 1 h. A mouse anti-calreticulin ER marker was used as a non-permeabilized condition control. Cells were washed with PBS and incubated with a goat anti-rabbit Alexa flour 488 (IgG H + L) or anti-mouse Alexa flour 647 (IgG H + L) antibodies (Abcam) for 1 h. Cells were washed and analyzed on a FlowSight^®^ Imaging Flow Cytometer (Merck, Kennborough, NJ, USA).

### 2.7. Co-Immunoprecipitation

HEK293T cells were co-transfected with pCAGGS_ORF3-FL or-Trnc and pCAGGS_S_AV12_. At 24 hpt, transfected cells were washed once with cold PBS. Cells were lysed with PierceTM lysis buffer (Thermo Scientific) supplemented with protease inhibitor cocktail (Thermo Scientific). Cell lysates were cleared by centrifugation at 14,000 times gravity (× *g*) for 5 min at 4 °C. Cleared lysates were then incubated with PierceTM anti-myc agarose (Thermo Scientific) with gentle rocking overnight at 4 °C. Immunoprecipitates were washed thrice with tris buffered saline and tween 20 (TBST;25 mM Tris-HCl, 0.15 M NaCl, 0.05% tween 20, pH 7.2) and eluted in sample buffer followed by sodium dodecyl sulfate polyacrylamide gel electrophoresis (SDS-PAGE) and Western blot.

### 2.8. Virus Titration

VeroE6-APN cells were grown in 6-well plates and inoculated with 10-fold serial dilutions of the recombinant PEDV_AV12_. At 24 hpi, infected cells were fixed with 80% cold acetone for 10 min and washed twice with PBS. Blocking solution containing 10% FBS and 1% BSA was added and incubated at room temperature for 1 h with gentle agitation. After blocking, cells were incubated with mouse anti-PEDV N antibodies (Medgene, Brookings, SD, USA) and goat anti-mouse IgG alkaline phosphatase antibodies (Abcam). Syncytium forming unit was examined based on color formation after the addition of 1-Step™ NBT/BCIP Substrate Solution (Thermo Scientific).

### 2.9. Statistical Analysis

GraphPad Prism 5.0 (GraphPad Software, La Jolla, CA, USA) was used for statistical analyses. The differences in mean values of virus titer between groups were analyzed by two-way ANOVA. All results were represented as means ± standard error of means; *p*-values of <0.05 were considered statistically significant.

## 3. Results

### 3.1. Generation and Characterization of Recombinant PEDV Bearing Tagged-ORF3

**Figure 1:**
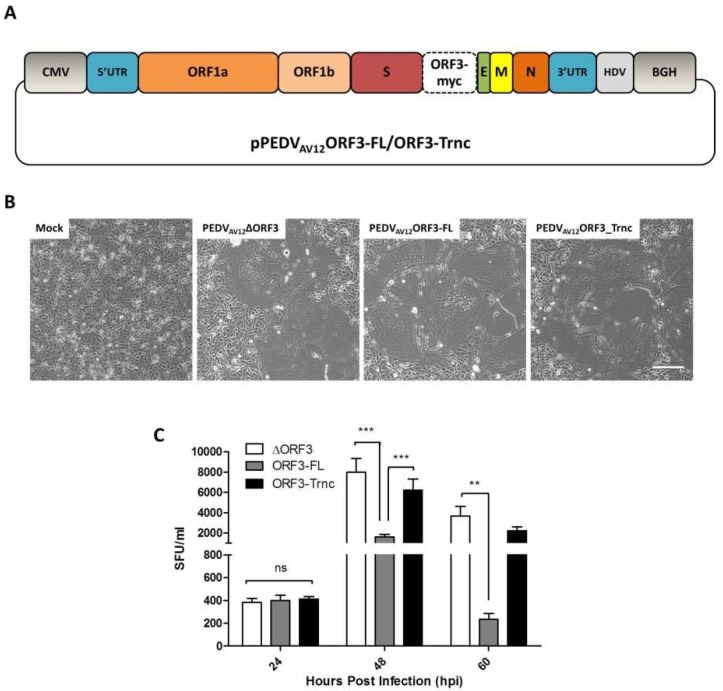
Generation of porcine epidemic diarrhea virus AVCT12 (PEDV_AV12_) infectious clones and growth kinetics of PEDV_AV12_ carrying myc-tagged ORF3. (**A**) schematic representation of PEDV_AV12_ infectious clone carrying myc-tagged ORF3 including the full length (ORF3-FL) or truncated ORF3 genes (ORF3-Trnc); (**B**) the PEDV_AV12_ infectious clones (pPEDV_AV12__ORF3-FL and-Trnc) were transfected into VeroE6-APN cells. At 48 hpi, syncytium formation was detected in the transfected VeroE6-APN cells. pPEDV_AV12_ with deletion of ORF3 gene (∆ORF3) was used as a control. Scale bar is 100 μm; (**C**) the reverse genetics-derived PEDV_AV12_-∆ORF3, ORF3-FL and-Trnc were inoculated onto VeroE6-APN cells at the multiplicity of infection (MOI) of 0.2. The viruses were harvested at 24, 48 and 60 hpi and subjected to syncytia forming unit assay (SFU) for virus titration. Statistical analysis was performed by using the two-way ANOVA method; error bars represent the means ± standard error of means of virus titers. ns; no statistical significance, ** *p* < 0.01 and *** *p* < 0.001.

Previous work with recombinant PEDV carrying various forms of ORF3 has revealed a role for ORF3 in PEDV replication [7]. However, due to the lack of effective ORF3 antibodies, we could not evaluate the expression and subcellular localization of ORF3 in infected cells. We thus attempted to generate recombinant PEDV_AV12_ carrying the codon-optimized ORF3-FL and-Trnc carrying myc-tag at the C-terminal as depicted in Figure 1A. The recombinant viruses, PEDV_AV12__ORF3-FL and-Trnc, could be propagated in VeroE6-APN cells with massive syncytium formation (Figure 1B). To confirm the presence of the ORF3 gene, we isolated viral RNAs of each recombinant virus and verified the sequence. Notably, both viruses contained the expected ORF3-myc with up to two amino acid mutations detected in some variants of ORF3-Trnc protein (Figure S1). To test whether the additional myc-tag affected the viral growth, we compared the viral growth kinetics between the reverse genetics-derived PEDV_AV12_ encoding untagged ORF3 and myc-tagged ORF3. We found that both viruses could efficiently infect and induce massive syncytium formation in VeroE6-APN cells with titers obtained from cells infected with each virus exhibiting no statistical difference (Figure S2).

We then examined PEDV_AV12__ORF3s replication in VeroE6-APN cells. When each virus was inoculated onto VeroE6-APN cells and harvested at 24, 48 and 60 hpi, we found that titers of all viruses were comparable early after infection, but different growth characteristics could be noted at the later time point with the PEDV_AV12__ORF3-Trnc titer significantly higher than that of the PEDV_AV12__ORF3-FL (Figure 1C).

**Figure 2:**
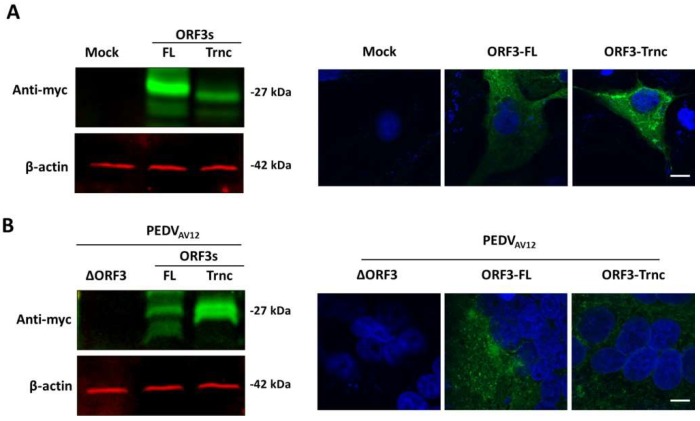
ORF3-myc protein expression in transfected (**A**) and infected cells (**B**). Cell lysates from transfected and infected cells were harvested and analyzed by Western blot analysis. Mock-transfected cells or PEDV_AV12_ _∆ORF3-infected cells were used as controls (**left panels**). VeroE6-APN cells were transfected with the plasmid expressing ORF3s-myc or infected with the recombinant viruses. ORF3-myc protein was probed by rabbit anti-myc antibodies, subsequently incubated with goat anti-rabbit IgG Alexa flour 488. Nuclei were stained with Prolong Gold Antifade Mountant with 4’,6-diamidino-2-phynyllindole (DAPI) and analyzed by confocal microscopy (**right panels**). Scale bars are 10 μm.

We subsequently examined the expression of ORF3 in the context of transfection and infection. To this end, VeroE6-APN cells were transfected with pCAGGS expressing the full-length and the truncated ORF3 or infected with the recombinant viruses. Recombinant virus lacking ORF3 (PEDV_AV12__∆ORF3) was also included as a control. As shown in Figure 2A, the ORF3-myc tag protein migrated to an expected molecular mass at ~27 kDa in both transfected and infected cells (Figure 2A,B, Left panel). However, the observed ORF3 protein bands expressed in transfected and infected cells appeared in different patterns. While the expressed ORF3s protein displayed a single band in transfected cells, additional bands were detected in infected cells (Figure 2B, Left panel). We surmised that the observed Western blot expression patterns of each ORF3 protein in infected cells are distinct and might not be relevant to other viral proteins and the PEDV growth as indicated by no correlation between the amount of S and ORF3s proteins in infected cells (Figure S3). Further analysis by immunofluorescence assay also revealed that the ORF3 proteins are predominantly localized in the cytoplasm of transfected and infected cells (Figure 2A,B).

### 3.2. Subcellular Localization of PEDV ORF3 Protein

**Figure 3:**
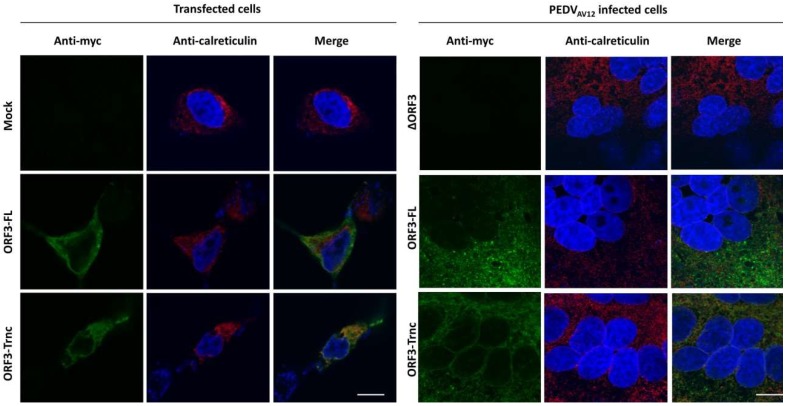
Subcellular localization of ORF3 in the endoplasmic reticulum (ER) of transfected and infected VeroE6 cells stably expressing porcine aminopeptidase N (VeroE6-APN). VeroE6-APN cells were transfected with pCAGGS_ORF3s-myc (ORF3-FL or-Trnc) (**left panel**) or infected by the PEDV_AV12__∆ORF3 and-ORF3s-myc (ORF3-FL or-Trnc) (**right panel**). Cells were stained with rabbit anti-myc and mouse anti-calreticulin ER marker as primary antibodies and goat anti-rabbit IgG Alexa flour 488 and-mouse IgG Alexa flour 647 as secondary antibodies. Nuclei were stained with Prolong Gold Antifade Mountant with DAPI. Co-localization of PEDV ORF3 protein with the ER marker was analyzed by confocal microscopy. Scale bars are 10 μm.

**Figure 4:**
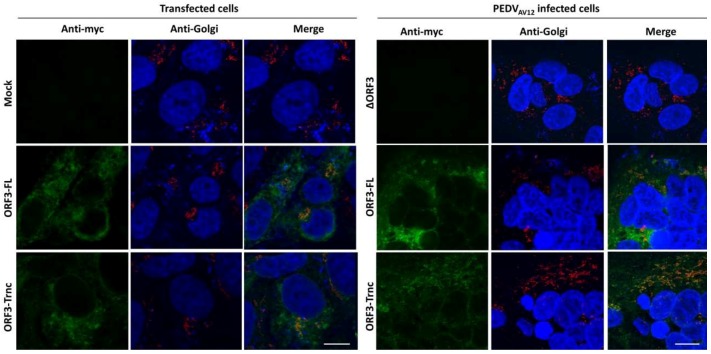
Subcellular localization of ORF3s in the Golgi apparatus of transfected and infected VeroE6-APN cells. VeroE6-APN cells were transfected with pCAGGS_ORF3s-myc (ORF3-FL or-Trnc) (**left panel**) or infected by the PEDV_AV12__∆ORF3 and-ORF3s-myc (ORF3-FL or-Trnc) (**right panel**). Cells were stained with rabbit anti-myc and mouse anti-58K Golgi marker as primary antibodies and goat anti-rabbit IgG Alexa flour 488 and-mouse IgG Alexa flour 647 as secondary antibodies. Nuclei were stained with Prolong Gold Antifade Mountant with DAPI. Co-localization of PEDV ORF3 protein with the Golgi marker was analyzed by confocal microscopy. Scale bars are 10 μm.

We next sought to determine whether the ORF3 protein is present in specific cytosolic compartments. Subcellular localization of ORF3 was first predicted in silico by WoLFPSORT program (https://www.genscript.com/wolf-psort.html), and results revealed that the protein has 12–17% identity with the integral membrane proteins such as V-type proton ATPase 16 kDa proteolipid subunit 2/3) and 12% identity with Olfactory receptor 13C9 located at the lysosome and plasma membrane. Interestingly, the ORF3 protein was expected to be in the endoplasmic reticulum (ER; 44%), Golgi apparatus (Golgi; 11%) and vesicle of the secretory pathway (11%). To assess the subcellular localization of the ORF3 protein in ER and Golgi, we performed confocal immunofluorescence staining of cells transiently expressing ORF3 or PEDV-infected cells. Investigation at a lower magnification was also performed to present a syncytium boundary in infected cells (Figures S4 and S5). Analysis of co-localization of ORF3 proteins demonstrated that the ORF3-FL and-Trnc are partially localized in the ER (Figure 3) and Golgi (Figure 4) with no statistical difference of PCC values between groups (PCC ± SD; ER: ORF3-FL = 0.48 ± 0.20, ORF3-Trnc = 0.76 ± 0.11 and Golgi: ORF3-FL = 0.44 ± 0.14, ORF3-Trnc = 0.51 ± 0.15, Figure S6). However, there was a significant decrease in co-localization of ORF3-FL with the ER and Golgi markers as compared to those from ORF3-Trnc in infected cells (PCC ± SD; ER: ORF3-FL = 0.12 ± 0.07, ORF3-Trnc = 0.46 ± 0.12 and Golgi: ORF3-FL = 0.27 ± 0.09, ORF3-Trnc = 0.65 ± 0.05, Figure S6), suggesting a distinct biological characteristics of the ORF3 variants during virus infection. Notably, we tested that localizations of ORF3 in the ER and Golgi compartments are not due to the extra C-terminal myc-tag by replacing the myc with Flag tag at the C-terminal region. The results showed that Flag-tagged ORF3 protein was also similarly localized in the ER and Golgi (Figure S7).

**Figure 5:**
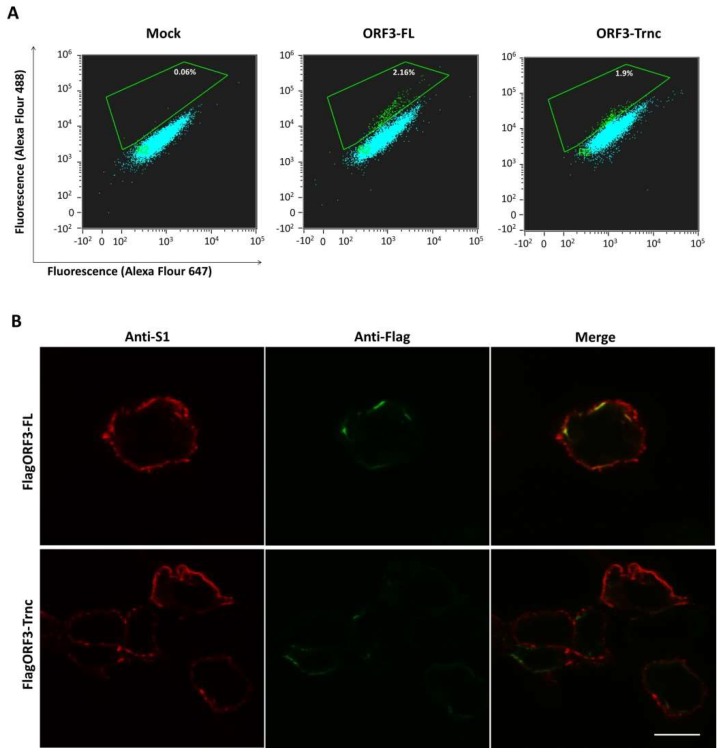
Subcellular localization of PEDV ORF3 protein on the plasma membrane. HEK293T cells were transfected with pCAGGS_FlagORF3s (FlagORF3-FL or-Trnc). At 24 hpt, cells were harvested and prepared under non-permeabilization condition; (**A**) the increased fluorescence signal against Flag-epitope was determined by flow cytometry. The percentage was calculated with respect to the whole cell population. Average transfection efficiency of HEK293T is approximately 80–90%; (**B**) the plasmid expressing S_AV12_ was co-transfected with pCAGGS_FlagORF3s in HEK293T cells grown on coverslips. Cells were prepared under non-permeabilization condition. Co-localization of PEDV FlagORF3 with S proteins was analyzed by confocal microscopy. Rabbit anti-Flag epitope or mouse anti-S1 antibodies and goat anti-rabbit IgG Alexa flour 488 or-mouse IgG Alexa flour 647 were used as primary and secondary antibodies, respectively. Scale bars are 10 μm.

Our results so far suggest that the ORF3 might utilize the ER–Golgi pathway for trafficking to the plasma membrane. To address this hypothesis further, we performed flow cytometry analysis to determine whether the ORF3 is expressed on the cell surface. We transfected HEK293T cells with the plasmid expressing ORF3-FL and-Trnc proteins bearing Flag-tag at the N-terminus. Notably, cells were harvested, fixed and analyzed in non-permeabilization condition. As depicted in Figure 5A, the increased signal could be detected on the surface of cells expressing pCAGGS_Flag-ORF3-FL or-Trnc at 2.16 and 1.9%, respectively. Nevertheless, the expression of ORF3 on the cell surface was relatively lower than those expressing S protein, which showed an increased signal at 8% (Figure S8). Intriguingly, when we performed the immunofluorescence analysis of cells co-expressing ORF3 and S, we observed that both proteins were co-localized at the plasma membrane (Figure 5B), which points to the possibility that both proteins might be associated with each other.

### 3.3. Localization and Interaction of PEDV ORF3 with S Proteins

**Figure 6:**
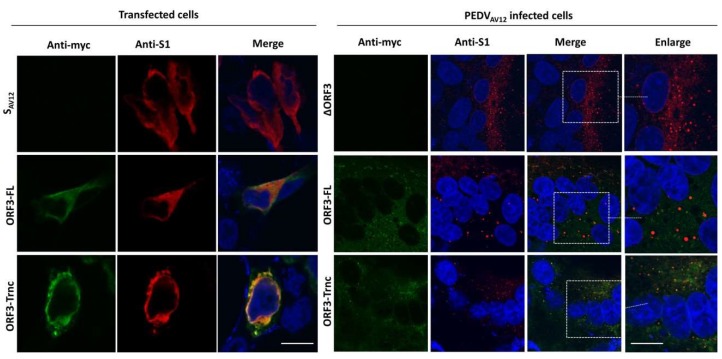
Co-localization of the ORF3 and S proteins in transfected and infected cells. HEK293T cells were co-transfected with pCAGGS_ORF3s-myc (ORF3-FL or-Trnc) and pCAGGS_S_AV12_ (**left panel**) or infected by the PEDV_AV12_ _ORF3s-myc (ORF3-FL or-Trnc) (**right panel**). The PEDV_AV12__∆ORF3 was used as a control. Cells were stained with rabbit anti-myc and mouse anti-S1 antibodies followed by incubation with goat anti-rabbit IgG Alexa flour 488 and-mouse IgG Alexa flour 647 antibodies. Nuclei were stained with Prolong Gold Antifade Mountant with DAPI. Co-localization of PEDV ORF3s with S proteins was analyzed by confocal microscopy. Scale bars are 10 μm.

To further investigate the possible interaction between ORF3 and S protein, we co-transfected pCAGGS_ORF3-FL or-Trnc and pCAGGS_S_AV12_ and analyzed transfected cells by immunofluorescence assay under permeabilization conditions. As shown in Figure 6, both ORF3-FL and-Trnc were localized with S_AV12_ in the cytoplasm with the ORF3-Trnc appearing more specifically associated with S_AV12_ particularly at the perinuclear area. Likewise, a similar pattern was noted in the context of infection as vesicle-like compartments containing S_AV12_, and ORF3 proteins could be detected (Figure 6). Additionally, co-localization of ORF3-Trnc with S_AV12_ was more concentrated and localized together in the vesicles (Figure 6) at a higher PCC value than the ORF3-FL in infected cells (PCC ± SD; ORF3-FL = 0.24 ± 0.07, ORF3-Trnc = 0.53 ± 0.04), Figure S6). Of note, mean PCCs to assess ORF3-S localization are shown in Table S2. To further determine whether ORF3 could be co-localized with the S protein derived from other PEDV strains, we overexpressed ORF3 together with the S protein from field-isolated PEDV in G2b genogroup (S-G2), which is substantially distinct from the G1 genogroup like S_AV12_ [13,14]. Similarly, ORF3 was found co-localized with S-G2 in the same manner as those observed in S_AV12_ (Figure S9).

**Figure 7:**
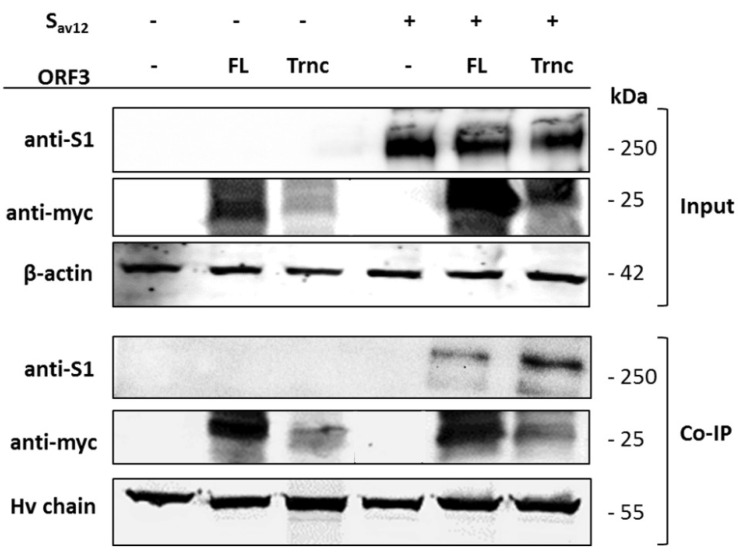
Co-immunoprecipitation of ORF3s and S proteins. HEK293T cells were transfected with pCAGGS plasmids expressing ORF3 (FL and Trnc) and S_AV12_. Cells were harvested at 24 hpt and subjected for incubation with anti-myc agarose beads. The pulled-down proteins were stained with rabbit anti-myc and mouse anti-S1 antibodies specific for ORF3-myc and S proteins. β-actin and IgG-heavy chain (Hv-chain) were used as loading controls.

To determine whether ORF3 interacts with S, we performed co-immunoprecipitation in cells transiently expressing ORF3 and S. As shown in Figure 7, both ORF3 variants could be pulled down with the S protein. In line with the immunofluorescence data, more S protein could be pulled down when co-expressed with the ORF3-Trnc. These results collectively indicate that ORF3 and S could interact with each other.

## 4. Discussion

We previously demonstrated that the ORF3 protein from different PEDV strains could have a distinct regulatory function on virus replication. Specifically, the naturally-occurring amino acid deletion could abolish the suppressive effect of some ORF3 variants on virus replication in vitro [7]. In this study, we synthesized the full-length ORF3 gene carrying C-terminal myc-tag (ORF3-FL) as a representative of the wild-type ORF3. To evaluate the effect of the amino acid deletion, we constructed the truncated ORF3 (ORF3-Trnc). In addition, we employed reverse genetics to generate recombinant PEDV carrying ORF3-myc without disturbing the virus growth and were able to follow ORF3 subcellular localization in infected cells. It should be noted that, while the amino acid substitution or deletion on ORF3-FL gene were not detected upon multiple passages, random amino acid substitution could be detected in some selected clones of ORF3-Trnc.

The ORF3 protein of PEDV has been predicted to have transmembrane domains and ion-channel function [5]. Similarly, accessory proteins from other coronaviruses such as U274 protein from Severe acute respiratory syndrome Coronavirus (SARS-CoV) [15] and ORF3 from Human pathogenic coronavirus NL63 (hCoV-NL63) [16] are also reported to have ion-channel activity. It is thus possible that these proteins may share common functions. Our results in this study, however, suggest that ORF3 may play multiple roles in addition to being ion channels during replication of PEDV in host cells. Consistent with this hypothesis, a recent study has demonstrated that ORF3 in cells stably expressing the protein was predominantly cytosolic [6]. In addition, cells expressing ORF3 exhibited a prolonged S phase, suggesting that ORF3 may be associated with the host’s cell cycle machinery [6]. Furthermore, our findings that the ORF3 protein expressed in transfected and infected cells displayed different patterns led us to speculate that the additional band in infected cells might be due to post-translational modifications that occurred during protein trafficking through the ER-Golgi compartments [15]. Indeed, the in silico analysis of ORF3 by the WoLFPSORT program predicts its cellular localization in ER, Golgi apparatus, and vesicle of the secretory pathway, which is confirmed by our immunofluorescence data. These results are actually in line with what reported previously about ORF3 [6,7] and other coronaviruses [16,17].

It is noteworthy that the ORF3-Trnc has a deletion of amino acids sequences ‘YCPLLYYCGAFLDATII’, resulting in the disruption of the transmembrane domain [5] and YxxΦ motif (x can be any residue, and Φ is a residue with a bulky hydrophobic side chain), a sorting signal for clathrin-mediated endocytosis [18,19]. The absence of YxxΦ motif within the SARS 3a protein was reported to increase retention of the ΔYxxΦ 3a protein in the Golgi apparatus, protein degradation and decreased protein expression on the cell surface [20]. Unlike the SARS 3a protein, our results showed that both ORF3-FL and-Trnc partially localized in the ER and Golgi compartments and could be transported to the cells’ surface, suggesting that the lack of YxxΦ motif in the truncated ORF3 protein might not be crucial for its subcellular trafficking through the ER–Golgi compartment.

Our observation that ORF3 and S are both expressed on the cell surface points to the possibility that these two proteins might interact with each other. Indeed, we showed that both ORF3-FL and-Trnc proteins were co-localized with the S protein. Interestingly, ORF3-Trnc displayed a more apparent association with the S protein particularly at the perinuclear membrane of transfected cells and the vesicle-like compartments in infected cells. Ultrastructural characterization in PEDV infected cells has revealed that the PEDV virus replication site was mainly at the perinuclear space where maturation of the virus particles was formed and packaged into the vacuoles through the vesicular pathway in the ER and Golgi compartments [21]. The fact that ORF3 were found co-localized with S protein at the perinuclear area and in the vesicular structures may suggest its contribution at this step of the replication cycle. It is also notable that other accessory proteins from other coronaviruses have been shown to interact with viral structural proteins such as S, M, and E proteins [15,16,20]. In addition, recent evidence has suggested a possible association between S and ORF3 to increase virus replication and virulence in nursing piglets. Recombinant PEDV viruses lacking ORF3 were of very low virulence despite the expression of S derived from a highly pathogenic variant. Interestingly, when the functional ORF3 was restored, the recombinant viruses bearing S from the pathogenic strain and ORF3 displayed increased virulence [22]. These data, therefore, suggest that S and ORF3 may work in concert to regulate PEDV replication in vivo.

Although the exact mechanism(s) by which the ORF3 protein takes part in virus replication has not been clarified in the present study, evidence of ORF3 characteristics described here uncovered its unprecedented functions and association with structural proteins that may play a role in virus assembly and growth. It is noteworthy that while some variants of full-length ORF3 were shown to inhibit PEDV replication [8], some studies report that the protein could promote PEDV replication in vitro [5,6]. In this regard, we cannot definitively distinguish whether ORF3 functions in promoting or suppressing PEDV replication or what other viral proteins could contribute to the regulatory role of ORF3. Consequently, further work is required to determine precisely how ORF3 exerts its functions. Understanding these functions may, in turn, enable the development of new attenuated vaccines to prevent PED.

## Supplementary Materials

The following data are available online at http://www.mdpi.com/1999-4915/10/8/399/s1, Figure S1: Amino acid alignments of full-length and truncated ORF3 genes obtained from the reverse genetic PEDV_AV12_-ORF3-FL and-Trnc; Figure S2: Growth kinetics of the reverse genetics-derived PEDV_AV12_ encoding myc-tagged (ORF3-myc) and un-tagged ORF3s (ORF3-wt) in VeroE6-APN cells; Figure S3: ORF3-myc protein expression in infected cells; Figure S4: Subcellular localization of ORF3 in the ER of infected VeroE6-APN cells at low magnification; Figure S5: Subcellular localization of ORF3 in the Golgi in infected VeroE6-APN cells at low magnification; Figure S6 Calculation of the Pearson’s correlation coefficient determining co-localization of ORF3 with different organelle markers and S protein in transfected and infected cells; Figure S7: Subcellular localization of ORF3Flag in the ER and Golgi apparatus of transfected cells; Figure S8: Expression of S protein on the plasma membrane; Figure S9: Co-localization of PEDV ORF3s and SG2 proteins in transfected cells. Table S1: Codon-optimized ORF3 with myc-tag at the C-terminus; Table S2: Pearson’s Coefficient analysis.
