# Supplementary material for: The Accessory Protein ORF3 Contributes to Porcine Epidemic Diarrhea Virus Replication by Direct Binding to the Spike Protein
Source: Viruses. 2018 Jul 28;10(8):399. doi: 10.3390/v10080399 (PMC6115756; doi:10.3390/v10080399)
Supplement: Supplementary file 1 [file viruses-10-00399-s001.pdf]

Supplementary Tables

Table S1 Codon optimized ORF3 with myc tag at C-terminus.

**Nucleotide sequence**
ACGCGT**ACC***ATGTTCCTGGGACTGTTCCAATACACCATTGACACCGTCGTGAAGGACGTGTCCA*<br>*AAAGCGCTAATCTGAGCCTGGACGCTGTCCAAGAGCTGGAGCTCAACGTCGTGCCCATCAGACA*<br>*GGCCAGCAACGTGACCGGCTTCCTCTTTACCAGCGTGTTCATCTATTTTTTTGCCCTGTTCAAGG*<br>*CCTCCAGTTTGAGGCGGAATTACATCATGCTGGCCGCCAGATTTGCCGTCATTGTGCTGTACTGT*<br>*CCTCTGCTGTATTACTGTGGCGCCTTCCTCGATGCCACAATTATTTGTTGTACCCTGATCGGAAG*<br>*ACTCTGTCTGGTGTGTTTTTATAGCTGGAGGTATAAAAATGCCCTCTTTATAATCTTTAACACCA*<br>*CAACCCTGAGCTTCCTGAACGGCAAGGCTGCTTACTACGATGGCAAATCTATAGTGATTCTGGA*<br>*GGGGGGCGATCATTATATTACATTTGGGAACTCCTTCGTGGCTTTCGTGAGCAGCATCGACCTGT*<br>*ATCTGGCTATTAGAGGCAGACAAGAGGCCGATCTGCAACTCCTCAGAACTGTTGAGCTGCTCGA*<br>*TGGAAAGAAGCTGTACGTCTTTTCCCAACACCAGATCGTGGGCATCACAAACGCAGCTTTCGAC*<br>*AGCATTCAACTGGATGAGTATGCCACCATCAGCGAG***GAGCAGAAGCTCATTAGCGAGGAGGA**<br>***CCTG***TAAGCGGCCGC
**Amino acids sequence**
MFLGLFQYTIDTVVKDVSKSANLSLDAVQELELNVVPIRQASNVTGFLFTSVFIYFFALFKASSLRR<br>NYIMLAARFAVIVLYCPLLYYCGAFLDATIICCTLIGRLCLVCFYSWRYKNALFIIFNTTTLSFLNG<br>KAAYYDGKSIVILEGGDHYITFGNSFVAFVSSIDLYLAIRGRQEADLQLLRTVELLDGKKLYVFSQ<br>HQIVGITNAAFDSIQLDEYATISE***EQKLISEEDL***-

Note: Underline is referred to *Mlu*I and *Not*I restriction sites, respectively. **Bold** is referred to Kozak sequence (**ACC**). *Italic* is referred to codon-*optimized ORF3*. ***Italic-Bold*** is referred to myc-tag.

Table S2 Pearson's Coefficient analysis.

Co-localization of ORF3s with	Mean Pearson's Coefficient	
	**ORF3-FL**	**ORF3-trnc**
**Transfected cells**		
ER	0.48±0.20	0.76±0.11
Golgi	0.43±0.14	0.51±0.15
S	0.51±0.15	0.90±0.04
**Infected cells**		
ER	0.12±0.07	0.46±0.12
Golgi	0.27±0.09	0.65±0.05
S	0.24±0.07	0.53±0.04

Note: The Pearson's Coefficient values are represented as mean ± SD.

Supplementary figures

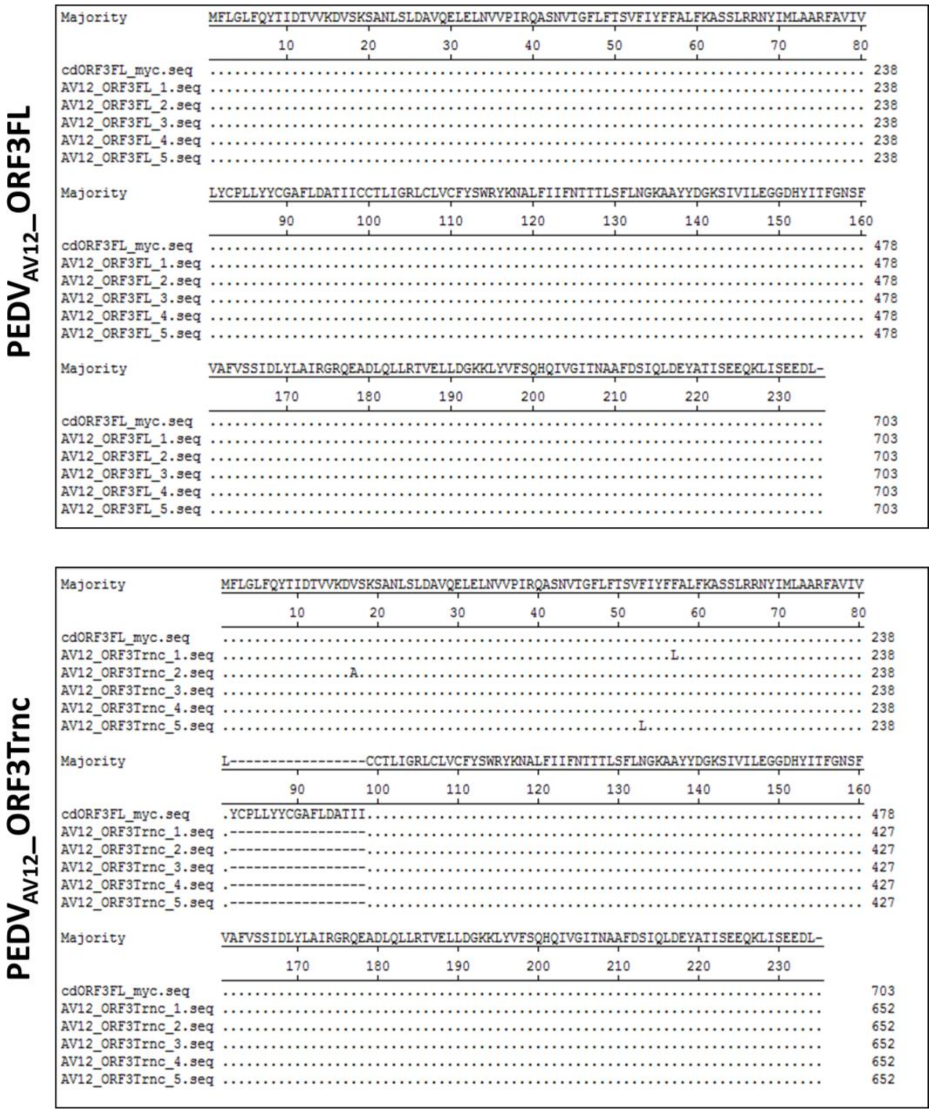

Figure S1 Amino acid alignments of full length and truncated ORF3 genes obtained from the reverse genetic PEDV$_{AV12}$-ORF3-FL and -Trnc. Viral RNA from the reverse genetic PEDV$_{AV12}$-ORF3-FL and -Trnc viruses was isolated and subjected for RT-PCR. The PCR products were cloned into pTZ5R/T vector and 5 independent clones were sent for sequencing to verify a presence of ORF3 gene.

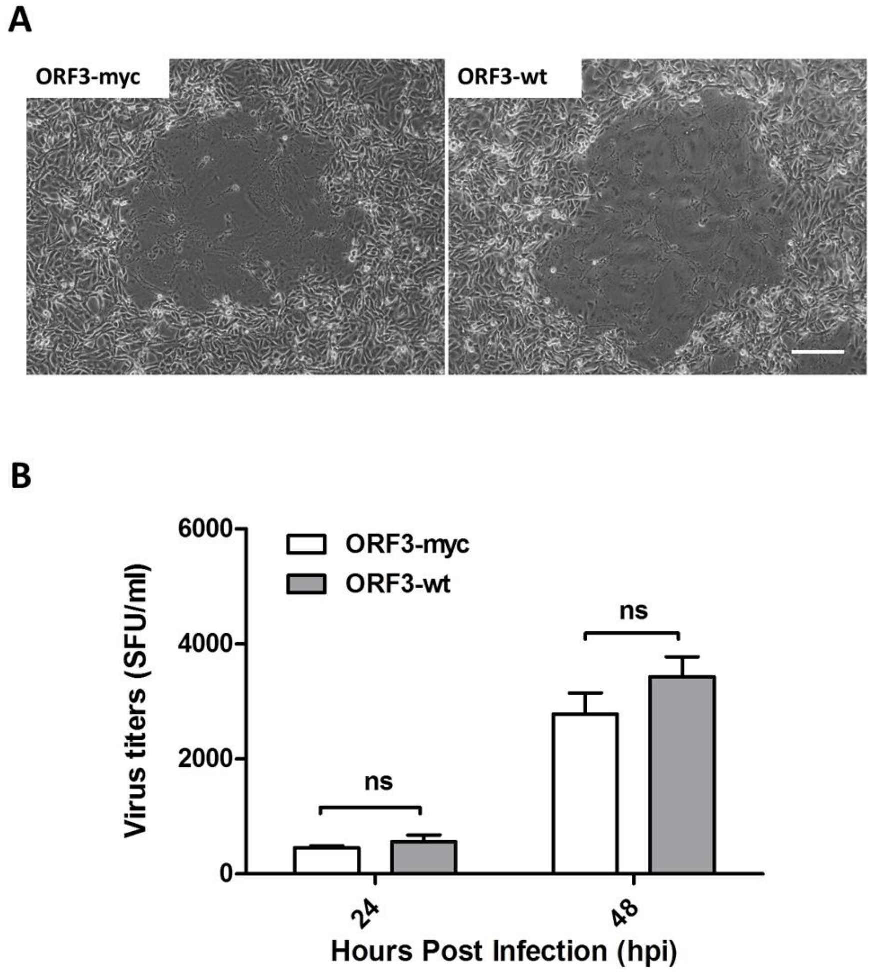

Figure S2 Growth kinetics of the reverse genetics-derived PEDV$_{AV12}$ encoding myc-tagged (ORF3-myc) and un-tagged ORF3s (ORF3-wt) in VeroE6-APN cells. The viruses were inoculated onto VeroE6-APN cells at MOI of 0.2. The viruses were harvested at 24 and 48 and subjected to syncytia forming unit assay (SFU) for virus titration. Scale bar is 100 μm. Statistical analysis was performed by using Two-way ANOVA method, Error bars represent the means ± standard error of means of virus titers. ns; no statistical significance, p>0.05.

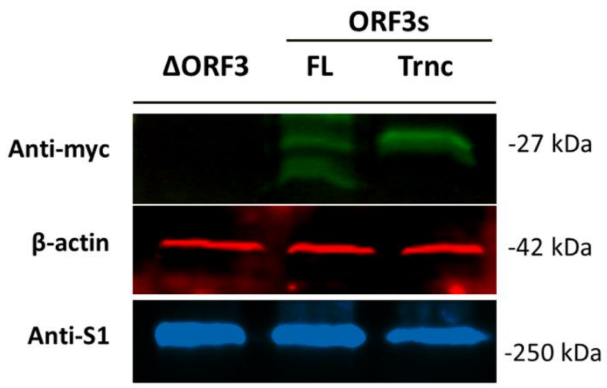

Figure S3 ORF3-myc protein expression in infected cells. VeroE6-APN cells were infected with the reverse genetic viruses, PEDV$_{AV12}$_ORF3-FL and -Trnc. PEDV$_{AV12}$ lacking ORF3 gene (ΔORF3)-infected cells were used as controls. At 24 hpt, cell lysates from infected cells were harvested and analyzed by western blot analysis. ORF3-myc and S proteins was probed by rabbit anti-myc and mouse anti-S1 antibodies, respectively. β-actin was used as a loading control.

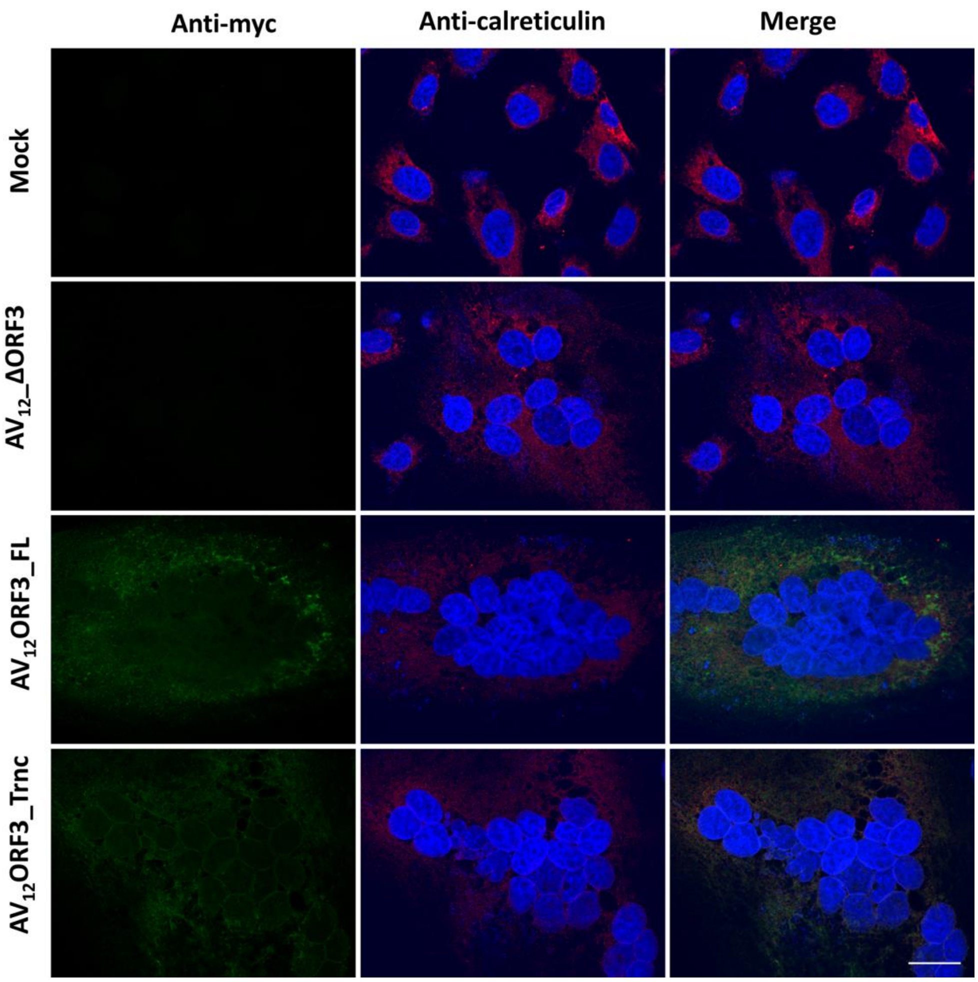

Figure S4 Subcellular localization of ORF3 in the ER in infected VeroE6-APN cells at a low magnification. VeroE6-APN cells were infected by the PEDV$_{AV12}$_ΔORF3 and -ORF3s-myc (ORF3-FL or -Trnc). Cells were stained with rabbit anti-myc and mouse anti-calreticulin ER marker as primary

antibodies and goat anti-rabbit IgG Alexa flour 488 and -mouse IgG Alexa flour 647 as secondary antibodies. Nuclei were stained with Prolong Gold Antifade Mountant with DAPI. Co-localization of PEDV ORF3 protein with the ER marker was analyzed by confocal microscopy. Scale bar is 20 μm.

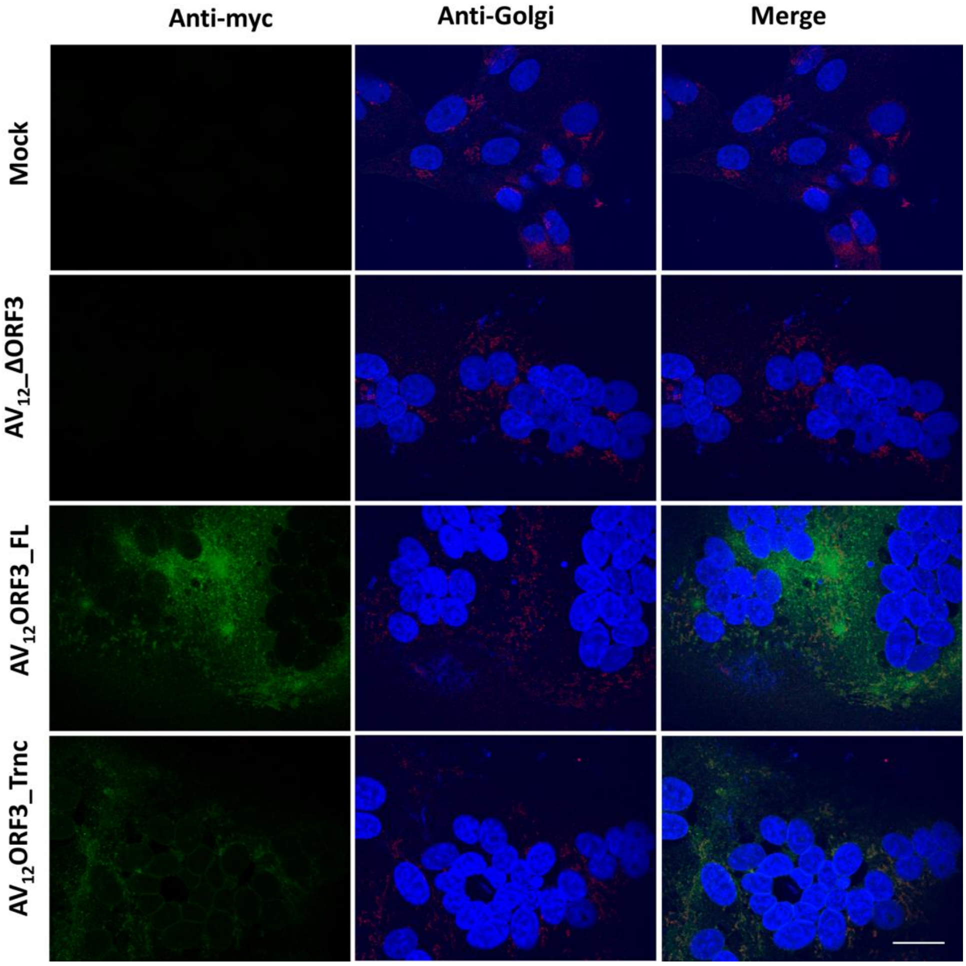

Figure S5 Subcellular localization of ORF3 in the Golgi in infected VeroE6-APN cells at a low magnification. VeroE6-APN cells were infected by the PEDV$_{AV12}$_ΔORF3 and -ORF3s-myc (ORF3-FL or -Trnc). Cells were stained with rabbit anti-myc and mouse anti-58K Golgi marker as primary antibodies and goat anti-rabbit IgG Alexa flour 488 and -mouse IgG Alexa flour 647 as secondary antibodies. Nuclei were stained with Prolong Gold Antifade Mountant with DAPI. Co-localization of PEDV ORF3 protein with the ER marker was analyzed by confocal microscopy. Scale bar is 20 μm.

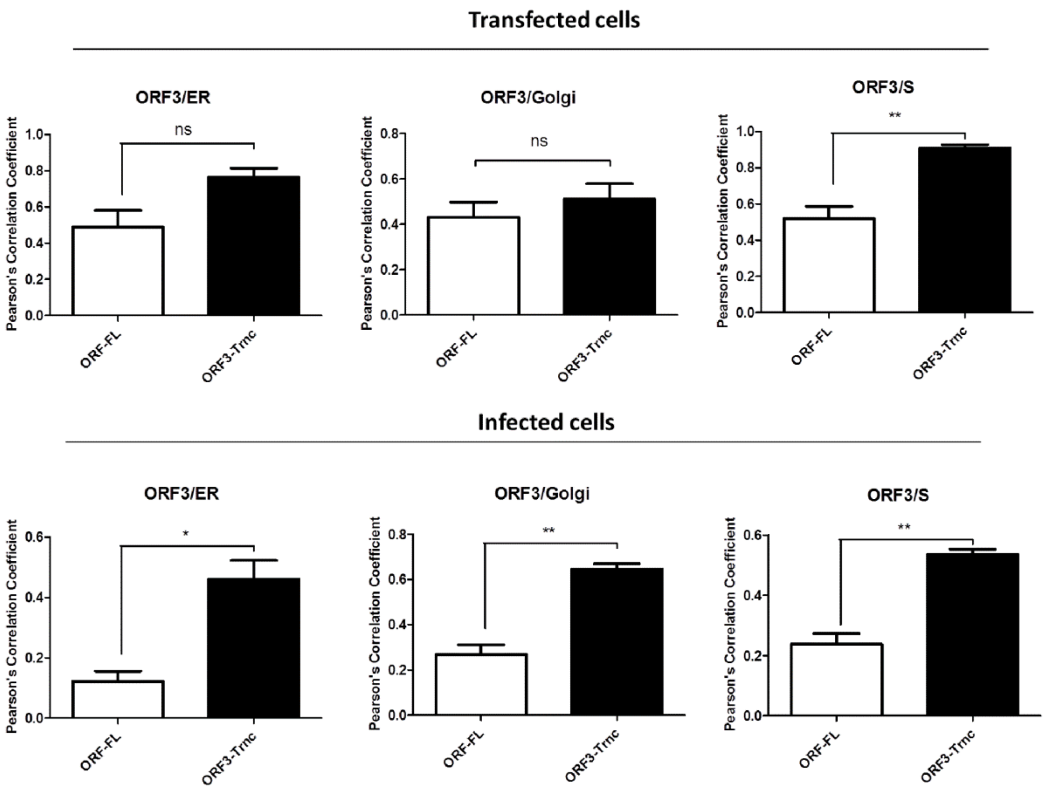

Figure S6 Calculation of the Pearson's correlation coefficient determining co-localization of ORF3 with different organelle markers and S protein in transfected and infected cells. Error bars represent the means ± standard deviation of the Pearson's correlation coefficients.
ns, no statistical difference, $p>0.05$. **, statistical difference, $p<0.01$.

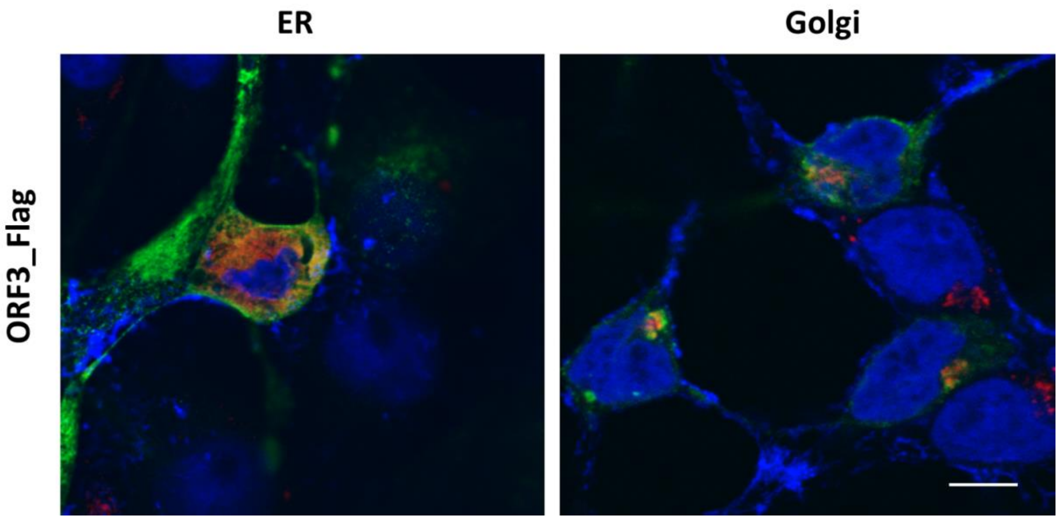

Figure S7 Subcellular localization of ORF3Flag in the ER and Golgi of transfected cells. Cells were transfected with pCAGGS_ORF3Flag and stained with rabbit anti-Flag antibody and either mouse anti-calreticulin ER marker or anti-58K Golgi marker as primary antibodies followed by goat anti-rabbit IgG Alexa flour 488 and -mouse IgG Alexa flour 647 as secondary antibodies. Co-localization of PEDV ORF3 protein with the ER and Golgi marker was analyzed under confocal microscopy. Scale bar is 10 μm.

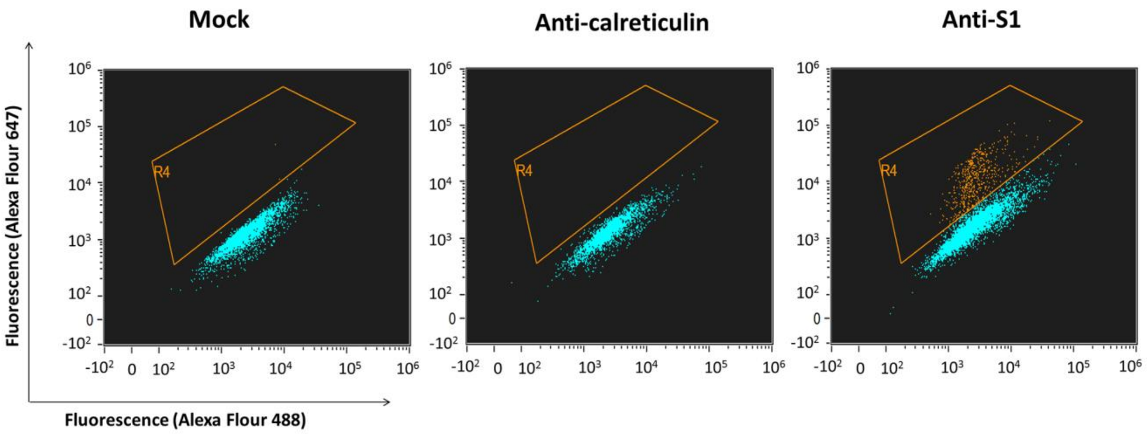

Figure S8 Expression of S protein on the plasma membrane. HEK293T cells were transfected with pCAGGS_FlagORF3s (FlagORF3-FL or -Trnc). To access a non-permeabilization condition, cells were stained with mouse anti-calreticulin ER marker. Mouse anti-S1 antibody was incubated with the cells transfected with pCAGGS_S$_{AV12}$ to examine S protein expression on the plasma membrane as a positive control. Goat anti- mouse IgG Alexa flour 647 was used as secondary antibody. The appearance of fluorescence signal against ER marker and S protein was determined by flow cytometry.

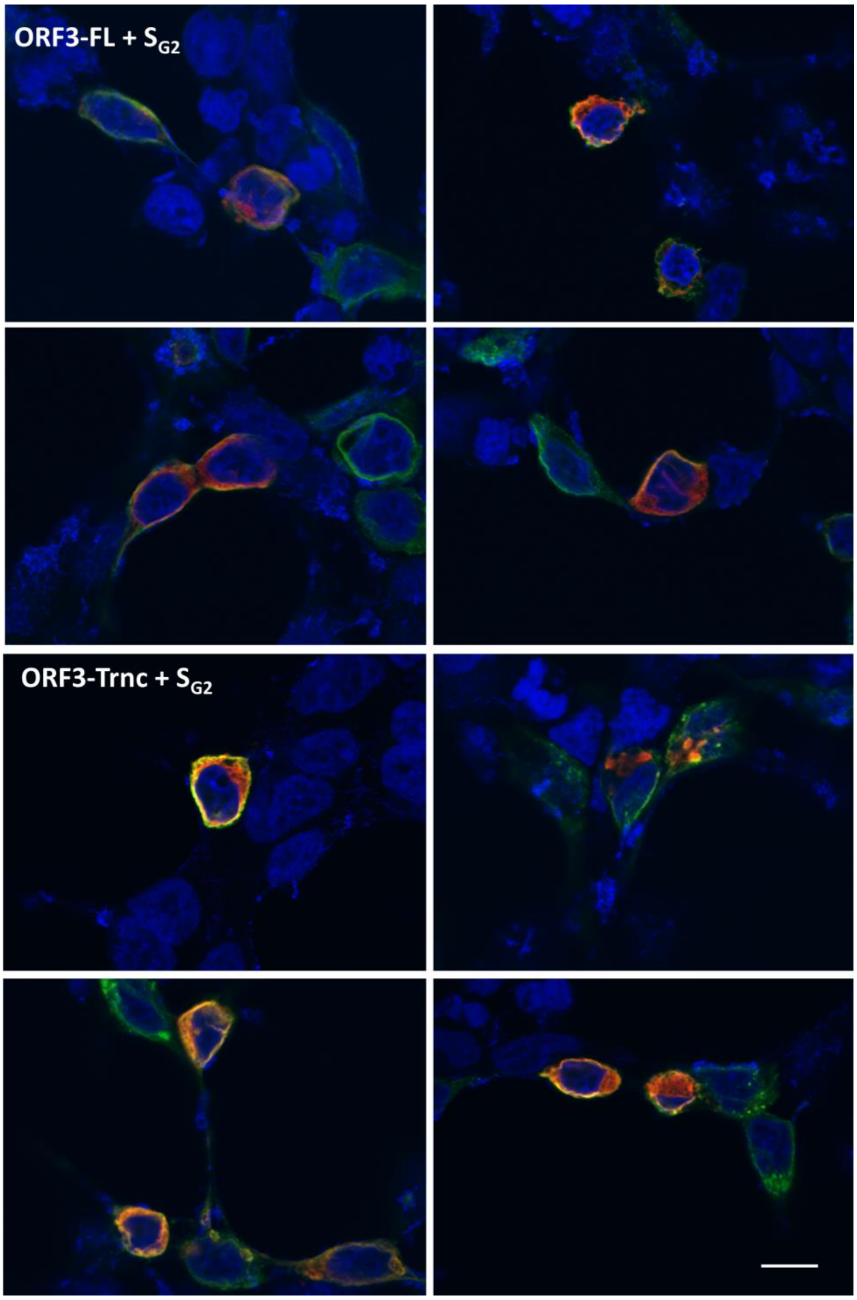

Figure S9 Co-localization of PEDV ORF3s and S_{G2} proteins in transfected cells. HEK293T cells were co-transfected with pCAGGS_ORF3s-myc (ORF3-FL or -Trnc) and pCAGGS_S_{G2} (left panel). Cells were stained with rabbit anti-myc and mouse anti-S1 antibodies followed by incubation with goat anti-rabbit IgG Alexa flour 488 and -mouse IgG Alexa flour 647 antibodies. Co-localization of PEDV ORF3s with S_{G2} proteins was analyzed under confocal microscopy. Scale bar is 10 μm.
